# Galvanising social innovation in gambling harms reduction: a process evaluation of a multi-component Community of Practice

**DOI:** 10.1177/17579759241293453

**Published:** 2024-11-24

**Authors:** Thomas Mills, Jo Evans, Catherine L. Jenkins, James Grimes, Paula Reavey, Jane Wills, Susie Sykes

**Affiliations:** 1 PHIRST South Bank, London South Bank University, UK; 2 Greater Manchester Combined Authority, UK; 3 Gambling with Lives, Sheffield, UK

**Keywords:** communities of practice, community-centred health promotion, gambling harms, VCFSE organisations, collaboration/partnerships, lived experience

## Abstract

**Background:**

Communities of Practice (CoPs) are increasingly used in health and non-health sectors globally. Evidence suggests that CoPs can support health promotion activities, but the research mainly encompasses formal, professional contexts: the role and contribution of CoPs in community-centred health promotion has not been explored. This paper presents a process evaluation of a CoP that aimed to facilitate social innovation among voluntary, community, faith and social enterprise (VCFSE) organisations. Hosted by a city-region government in England, VCFSE organisations were invited to join a CoP to enable the development and implementation of their ideas for addressing gambling harms.

**Methods:**

The process evaluation sought to develop mid-level programme theory for the use of CoPs in community-centred health promotion. Data collection consisted of 33 qualitative interviews with stakeholders, as well as project reporting. Data were organised using a framework approach which supported the construction of themes and a complex intervention model. The research team reflected on these to develop the programme theory.

**Results:**

The CoP facilitated the development of community-centred interventions for addressing gambling harms through a two-track process: first, a community of VCFSE staff was formed, whose understanding of gambling harms was nurtured through discussions led by people with Lived Experience; second, the CoP contributed to project development via collaboration, knowledge sharing and an integrated referral pathway, although project-level benefits were uneven. Learning was generated in community engagement, training, education, support and social campaigns.

**Conclusions:**

The findings confirm the combined effectiveness of a CoP, varied VCFSE projects and people with Lived Experience to co-create an evolving knowledge-base for a city-region government’s gambling harms reduction strategy. CoPs may therefore complement partnership working in community settings, although additional training support may be required in comparison with CoPs involving health professionals. The city-region government’s approach could be replicated in other emerging public health areas.

## Background

Communities of Practice (CoPs) aim to solve complex problems by facilitating collaboration and mutual learning (1–3). They feature three fundamental elements: a *domain of activity*, which facilitates group identity; a *community* linked by common interests in the domain; and a shared *practice area*, which encapsulates the language, ideas, frameworks and interventions developed or refined by members (2). The method is utilised by prominent international organisations, including the World Bank, World Health Organisation and European Commission, with the latter’s ‘Community of Practice Playbook’ providing guidance for ‘gathering, sharing and using’ of information and knowledge through CoPs (3).

The collaborative nature of CoPs aligns with longstanding health promotion interest and practice in multisectoral collaboration, networks and partnerships (4). Although these more general constructs can take on similar forms, CoPs have a more specific aim of generating, refining or coordinating knowledge (1,3). A recent evaluation of a CoP based in New South Wales, which aimed to support health promotion practitioners’ efforts to implement a healthy lunchbox programme, found that the CoP facilitated practice improvements via information sharing and collaboration: the authors concluded that CoPs may help address limitations of other, less data-driven forms of health promotion collaboration (5). In other health contexts, evidence suggests that CoPs can facilitate improvements in service performance, although gaps in understanding exist regarding how CoPs work in different types of settings (2,6,7).

Indeed, the research literature primarily focuses on CoPs involving health professionals in formal organisational contexts (2,6,7): hence, Hennein *et al*. highlight a need for research on CoPs for ‘lay health workers’ who do not have ‘formalized health professional education’ (2). This poses the question of a possible role of CoPs in community-centred health promotion, where the focus is involving and supporting community actors in efforts to improve health and wellbeing. Here, as with health promotion more generally, partnerships and networks are well-established (8). The processes underpinning these are often not the primary focus of research yet are useful to study to develop an understanding of the different forms of partnership and how these can be successfully implemented (4,9). In this paper, we explore the use of a CoP to support the commissioning, development and delivery of community-centred interventions in partnership with diverse voluntary, community, faith and social enterprise (VCFSE) organisations.

## A CoP for social innovation in gambling harms reduction

This paper presents a process evaluation of a CoP for galvanising social innovation among VCFSE organ-isations in response to the emerging public health issue of gambling harms (10). The CoP was implemented by a city-region government public health team as part of an initiative called ‘Communities Addressing Gambling Harms’ (CAGH). The CoP aimed to support the development of ‘social innovations’ (11) to define and address local need, but also to contribute to the evidence-base in community-centred gambling harms reduction (12).

The public health team, as commissioner-facilitators, invited VCFSE organisations to apply for funding by putting forward ideas for a gambling harms reduction project, with the process simplified to encourage organisations that were inexperienced in formal applications. Rather than commission the VCFSE organisations separately, commissioner-facilitators invited successful organisations to attend the CAGH CoP: 12 VCFSE projects completed the process.

Applying CoP terminology (1,2), the *domain of activity* was the emerging public health area of gambling harms while the *community* included commissioner-facilitators and VCFSE staff. The CoP’s *practice area* was community-centred health promotion oriented to addressing gambling harms, which the VCFSE staff were engaged in through the 12 projects. These 12 projects involved varied health promotion activities including gambling harms education in schools, community outreach with diverse ethnic communities, campaigns to create healthier environments (e.g. by ending gambling advertising in sports) and social support for people encountering gambling harms. It was anticipated that participation in the CoP would help VCFSE staff to develop and implement their ideas across these varied activities. A Lived Experience (LE) advisory panel was also set up alongside the CoP: panel members can be considered peripheral members of the community as they were not expected to attend all CoP sessions but were available for commissioner-facilitators and VCFSE staff to ask for advice and support if needed. The diverse contributions of people with LE to addressing gambling harms are explored elsewhere (13).

This process evaluation sought to understand how the CoP supported VCFSE staff’s efforts to develop and implement innovations at a project level. Process evaluations are a vital component of intervention research (14). The Medical Research Council’s guidance on complex intervention research highlights their role in developing programme theory for understanding how complex interventions work. Here, we adopted an iterative approach to theory development: an initial programme theory, supported by a logic model, was coproduced through four workshops involving the research team, commissioner-facilitators, senior CAGH advisors and two people with LE of gambling harms who were involved in CAGH. An abductive, process-oriented approach was then utilised to test and refine the programme theory, in line with established recommendations (14,15). A Public and Patient Involvement and Engagement panel consisting of three people with LE of gambling harms, who were all members of the LE advisory panel linked to CAGH, guided the research process.

## Methods

### Data collection

Data collection started six months into CAGH’s 18-month delivery period. Semi-structured interviews were carried out with three groups of participants who were recruited via purposive sampling: senior CAGH advisors, who included public health professionals working at local, regional and national scales (SCA = *n*-6), people with LE involved in the LE advisory panel (PLE = *n*-7) and VCFSE project staff, which included project leads and frontline staff (VPS = *n*-16). A semi-structured topic guide was developed based on a logic model that was developed as part of the evaluation design process (see Supplemental material file 1 online). Interviews were concentrated at the mid- and end-points of the CoP, aiming to generate insight into the processes through which the CoP shaped the VCFSE projects and project level learning at the end-point. Some participants were interviewed twice because they were deemed to have important insight on a topic of interest. The research team agreed to conclude the interviews based on their assessment of the ‘information power’ (16) of interview data. Interviews were characterised by high quality dialogue that gleaned rich and relevant data on the CoP while most stakeholders who directly participated in CoP sessions were interviewed: the sample included 11 out of 12 project leads who attended the CoP, as well as seven out of nine people with LE who supported it via the advisory panel; the outstanding three people did not reply to the research team’s emails. In total, 33 interviews were carried out by TM, CJ and PR, ranging from 40 min to 1 h 27 min.

‘Learning reflections’ reports were collected from the VCFSE projects with gaps completed through discussions between the evaluation team and VCFSE staff (see Supplemental file 2 for the template). These, along with projects’ initial proposals for joining CAGH, provided further data for analysis.

### Data analysis

Data analysis intertwined with data collection, writing and group theorisation. The initial coding framework was informed by the topic guide, with emergent codes and categories incorporated into this as the analysis progressed. The coding framework was applied to all data, including the ‘learning reflections’ reports, using NVIVO V12. Summaries of findings were written by TM and shared among the research team from the mid-point onwards, with group theorisation conducted. Once all data were collected, an initial set of themes and a complex intervention model (17) were derived by TM and JE through three 1.5 h analysis and theorisation meetings. These were then shared and discussed with the wider research team and refined through consensus discussion.

## Findings

Data pertaining to the role and contribution of the CoP within CAGH were organised into two themes:

- Theme 1 – The CoP: establishing a community of informed and enabled VCFSE staff;
- Theme 2 – The VCFSE projects: the varied practical utility of the CoP.

### Theme 1: The CoP: establishing a community of informed and enabled VCFSE staff

The CoP brought a community dimension to CAGH that was widely held to have added value, with seven CoP sessions held over the 18-month delivery period. VCFSE staff who attended CoP sessions reported many intangible benefits. These included fostering a sense of group identity among community members who shared common interests in finding out ways to address gambling harms. That the gambling harms domain was emergent and politically contentious appeared to be relevant as the CoP facilitated a sense, among some VCFSE staff, that they were part of a movement for change:

The CoP sessions . . . it feels like you’re part of a movement to change things. (Project report 11)

Community members said they were inspired by other projects and that it was both interesting and helpful to learn about what others were doing. For those VCFSE staff who experienced implementation challenges at a project level, moreover, it was reassuring to share these among community members during CoP sessions. This was important because, with so few organisations addressing gambling harms locally, it could be isolating and lonely for new entrant organisations:

It kind of relaxes everyone . . . It’s very reassuring . . . to hear that the same successes are there [and] the same barriers are there. (VPS4)

A more tangible benefit of the CoP was its impact on the awareness and understanding of gambling harms among VCFSE staff, many of whom were new to the domain. A needs assessment document, which combined statistical data with LE perspectives, was produced by commissioner-facilitators and discussed among the community, with some VCFSE projects referencing findings in project resources. The community also decided to invite people with LE to discuss their experiences of gambling harms and advise on how to talk about these and the people who suffer them. Issues with the labels of the ‘problem gambler’ and ‘responsible gambler’ were discussed. A VCFSE staff member welcomed these LE-led discussions on gambling harms narratives because they had been nervous entering the domain:

That’s something that I was very nervous about. When I first started . . . I was quite cautious of not wanting to offend people and getting it right. And there’s such a conflict in terms of what the right words are. (VPS8)

The VCFSE staff member adapted their language, based on what was discussed in CoP sessions:

Across all of my work, I would say, ‘gambling harms’ in every context. So ‘affected by gambling harm, either through addiction or as an affected other’: that’s how I would phrase it. (VPS8)

Commissioner-facilitators also learnt from CoP sessions, notably about how gambling harms were encountered locally and what to do to support the individual VCFSE projects. However, limited staff resources at the commissioner-facilitator level slowed the delivery of timely support to each individual project. Towards the end of the initial 18-month delivery period, commissioner-facilitators developed the CoP into a regional gambling harms network for supporting the sustainability of the VCFSE projects and to facilitate new VCFSE organisations into the domain. This followed the recommendations of community members:

I would recommend, to continue the momentum of the work done by all the organisations involved in the Community of Practice . . . an alliance should be created in order for the group to . . . carry on delivering the message of prevention of gambling harms. (Project report 7)

### Theme 2: The VCFSE projects: the variable practical impact of the CoP

The 12 VCFSE projects underwent a process of iterative design and delivery with staff’s initial project ideas evolving considerably. Commissioner-facilitators themed the projects towards the end of the 18-month delivery period to better understand the varied social innovations to emerge from this process. Key learning points from the projects are presented in Table 1, along with details of the number of projects involved in the social innovation theme (the 12 projects overlapped across themes), target groups and underpinning health promotion activities.

**Table 1.** Learning on social innovations for gambling harms.

Social innovation theme	Target group	Health promotion activities	Learning point
Community engagement – six projects	Diverse underserved communities and ethnic groups	- LE-led awareness-raising platforms - Community outreach via ‘Community Angels’ and group awareness activities - Community drama show - Social marketing campaigns - Social media campaigns - Local press coverage	*Community engagement initiatives can reach diverse undeserved communities and ethnic groups through LE-led awareness-raising platforms*
Training – five projects	Diverse community and professional actors with capability to intervene	- Training for community sports coaches, teachers, and drug and alcohol workers - Awareness-raising events - LE involvement to advise on language and imagery	*Training in gambling harms assessment, signposting and support is relevant to many people across the community, health and education sectors*
Education – three projects	Young people of school age and young adults, including at risk groups	- New educational materials with a focus on harmful products - Education delivery in popular community settings - Gambling harms incorporated into existing education programmes	*Education on harmful industry products and practices is engaging and avoids both moralising messages and stigmatising language*
Support – two projects	Anyone encountering GRH and needing support	- LE-led support, including individual and group options - National provider develops base in region - Local provider adapts support programme for domestic violence victims	*Community support organisations led by people with LE can provide accessible and person-centred support that complements NHS gambling clinics*
Social campaigns – one project	Decision-makers in sports organisations, sports fans and the general public	- Charter for ending gambling sponsorship - Meetings with community trusts of professional clubs - Social marketing campaigns - Website - Social media campaign - Message of support from the Mayor, published on YouTube	*Social campaigns for ending gambling sponsorship in sports can stimulate health promoting environmental changes despite a challenging commercial and policy/regulatory environment*

LE: Lived Experience; NHS: National Health Service

VCFSE staff differed in their views regarding the extent that the CoP had impacted beneficially on their individual project and thus stimulated the learning expressed in Table 1. In some cases, the CoP facilitated cross-project collaboration that meant host organ-isations’ pre-existing community assets could be utilised. For example, one educational innovation was underpinned by a collaboration involving two VCFSE organisations, with the first organisation developing and delivering the innovation (and utilising the organisation’s pre-existing expertise in education in the process) and the second providing access to a popular community setting, at which the education was delivered. The second VCFSE organisation also had links to statutory organisations that were utilised to raise awareness of the project:

[Our partner has] been very helpful, getting other councils and public health bodies . . . to help us spread the message out to further areas. So . . . being part of something like this makes it easier to make connections that you wouldn’t normally [have]. (VPS7)

Delivery of the educational intervention was still challenging initially yet knowledge sharing within a CoP session helped. A VCFSE staff member on a different project provided advice on how to generate more interest from schools:

It was good advice, and we then went away and changed the flyer that we used. We said that, although the focus is on gambling, we cover online safety, mental health, coping and online gaming . . . [That] is the value of those [CoP] meetings, because that was something we had not massively thought about: . . . we can be quite single [issue] focused. (VPS7)

A further contribution of the CoP at project level was providing a referral pathway for VCFSE organisations to refer people to the two support projects if they needed support for gambling harms. The emergence of this pathway had not been anticipated initially. A VCFSE staff member who received referrals via the pathway planned a ‘wraparound’ approach with another project:

I received two referrals . . . I am actually going to refer them to one of the other gambling people from the Community of Practice . . . One of them is . . . [suffering] financially . . . because of it, so I’m going to get into contact with her and look at getting her on that, and then maybe help with the domestic abuse side of it here. It’s a . . . wraparound approach . . . [It will be us who does] the counselling here if it’s something that they want to pursue, and we also have the contacts now to signpost. (VPS10)

Yet the CoP’s practical utility was questioned by two of the 12 projects: a support project and a social campaign project. It is notable that these social innovation themes featured the fewest projects, at two projects and one project respectively (see Table 1): the projects therefore had fewer projects with similar practice areas to share learning and collaborate with, which lessened the benefits of collective provisioning:

I think the CAGH Community of Practice perhaps did not prove to be as effective as anticipated because the programmes that were funded were so different. (Project report 3)

A further issue concerned the extent that LE-led discussions in early CoP sessions, regarding the importance of avoiding stigmatising language, had shaped project level narratives and resources. Some people with LE were concerned with some projects’ ongoing use of stigmatising language which echoed the research team’s observation that one VCFSE project’s initial project resources featured highly stigmatising notions of the ‘problem gambler’. Commissioner-facilitators responded by commissioning freely available training support for VCFSE organisations. The issue is indicative of challenges moving away from stigmatising language in community settings, which the CoP could only partially overcome:

Convincing all the organisations involved, who only meet once a month, who have got a million other things going on, who are trying to get the sessions up-and-running, whatever: to really get into the detail of language is difficult. And I saw [reference to VCFSE project], who are involved in this Community of Practice, still say ‘problem gambler’, ‘problem gambling’. (PLE1b)

## Discussion

CoPs are increasingly common globally, being pro-moted by prominent international organisations such as the World Bank, World Health Organisation and European Commission (2,3). This paper has explored the role and contribution of CoPs to health promotion partnerships involving VCFSE organisations, which has hitherto been under explored (2), through the example of the CAGH intervention. This is important as VCFSE organisations are widely recognised, in community-centred health promotion, as vital for utilising the community assets that nurture health and wellbeing (18,19).

The CAGH CoP enabled a process of social inno-vation which generated learning on community-centred gambling harms interventions. Based on the themes above, our programme theory posits a distinct role and contribution for CoPs in partnerships with VCFSE organisations:

- CoPs can galvanise social innovation in emerging and politically contentious health promotion domains by, first, forming a supported community of VCFSE staff, whose understanding of the domain is nurtured through LE-led narrative discussions (see Theme 1); then, second, facilitating cross-project collaboration, knowledge sharing and referrals across diverse VCFSE projects which proceed through iterative design and delivery (see Theme 2).
- This two-track process, when successfully implemented, generates learning on social innovations (see Table 1) that may inform scale-up efforts.

**Figure 1.**
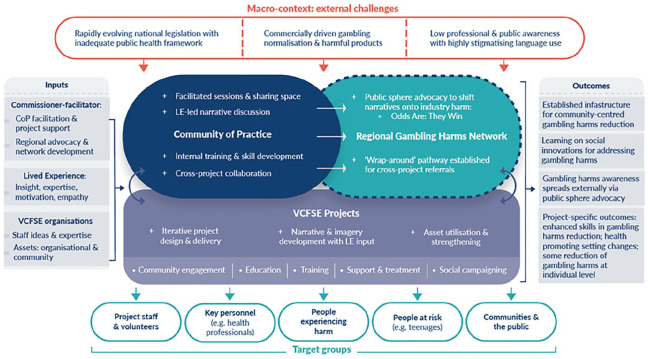
The Communities Addressing Gambling Harms initiative. The Community of Practice (CoP) is represented by the blue oval which merges into the turquoise oval to reflect its evolution into a regional gambling harms network to scale up the learning generated at the project level. Underneath the CoP are the 12 voluntary, community, faith and social enterprise (VCFSE) projects which proceed through a process of iterative design and delivery. The various target groups of Communities Addressing Gambling Harms (CAGH) are situated at the bottom of the model, starting with VCFSE project staff and volunteers who, after learning about gambling harms in the CoP, push out to raise awareness of gambling harms or directly work with other target groups. CAGH outcomes (listed to the right of the model) include established infrastructure for ongoing community-centred gambling harms reduction, learning on social innovations, the outcomes linked to the 12 VCFSE projects and enhanced awareness and understanding that is stimulated externally through public sphere advocacy. Such wider impact was an anticipated consequence of the combined advocacy of the 12 projects and a concurrent Lived Experience (LE)-informed social marketing campaign, called ‘Odds Are: They Win’.

Our complex intervention model provides a diagrammatical representation of the CoP within the CAGH intervention (see Figure 1). This highlights how CAGH sought to counter commercially driven gambling normalisation and harmful products partly through the dissemination of counter-industry narratives. This ‘counter-industry’ component, which we explore elsewhere (13,20), has not been explored directly here because we consider the CAGH model to be potentially relevant for health promotion domains that do not have a commercial determinants angle. The politically contentious nature of the gambling harms domain appears to have added to the importance of collective commissioning through a CoP as it enabled and supported VCFSE staff. As such, the CAGH model may be relevant to other politically contentious or stigmatised health issues that require collective protections and support, as well as a first-stage of learning about appropriate language prior to project delivery. For example, it may be relevant for amplifying the voices of refugees and asylum seekers across community services, or the voices of trans and non-binary people.

Important research and practice implications follow.

We suggest that CoPs are complementary to the many methods, tools and approaches that make up community-centred health promotion. An explicit focus on generating learning on new or enhanced forms of ‘practice’ makes CoPs distinct from other forms of collaboration and partnership (8).

In the CAGH case, commissioner-facilitators aimed to scale-up the successful innovations presented here (see Table 1) by inviting other VCFSE organisations to join the community and build on and refine learning on ‘what works’. Taking one example from CAGH’s five social innovations themes, the community engagement projects established the impact of LE-led awareness-raising events at community settings, such as places of worship. Bringing in more faith organisations to collaborate with those already involved in CAGH could lead to similar events being convened across the region. This amplifies the common partnership outcomes of increased knowledge, skills and confidence (8), as VCFSE staff are enabled to co-create an evolving knowledge-base for the community arm of a city-region government’s gambling harms reduction strategy.

We therefore agree with Barnes *et al*. (5) that CoPs can strengthen the many forms of health promotion collaboration that exist. In our view, it is advisable to align partnerships or networks that have a knowledge orientation with CoPs, as this makes accessible relevant CoP theory, guidance documents and evaluation frameworks designed to support this (1,3). We conclude by noting some challenges that may be unique to CoPs in the VCFSE sector.

The well-established finding that CoPs can facilitate impact via collaboration and learning among health professionals (2,6,7) is confirmed here, among VCFSE staff. However, stigmatising language was identified at the project-level in the early stages, which may reflect how the CAGH CoP served to initiate VCFSE staff into what was for many a new domain. This prompted commissioner-facilitators to commission additional LE-led training for VCFSE staff. In addition, whereas CoPs in formal health settings typically feature a shared practice area in the form of a standardised work stream or service, the CAGH CoP consisted of varied practice areas linked to different social innovation themes (i.e. community engagement, training, education, support and social campaigns). This posed challenges with two of 12 projects reporting that the CoP had not been of practical value. Commissioning groupings of projects that share a practice area is, therefore, advisable.

## Limitations

This paper presents a qualitative case study conducted in a single city-region area in England. A limitation of this approach is that it can lack transferability although we have utilised ‘thick description’ through the thematic findings to partially offset this issue. While the study’s process-oriented focus identified key learning points from the VCFSE projects, more detailed, project level research is required to develop and evaluate the social innovations: this would generate more definitive insight into the contribution that community-centred health promotion can make to efforts to address gambling harms than we have provided here.

## Conclusion

This study suggests that CoPs, which are increasingly used in health and non-health sectors globally, can contribute positively to health promotion partnerships in community settings. The CAGH CoP generated learning on ‘what works’ in community-centred health promotion with crucial inputs from people with LE of gambling harms, who shaped VCFSE staff’s understandings of the problem being addressed. The interventions were diverse and reflective of health promotion strategies in community engagement, training, education, support and social campaigns, with the CoP evolving into a network for supporting regional scale-up. This approach could be replicated in other emerging and politically contentious public health areas which require a community-centred health promotion response.

## Supplemental Material

sj-docx-1-ped-10.1177_17579759241293453 – Supplemental material for Galvanising social innovation in gambling harms reduction: a process evaluation of a multi-component Community of Practice Supplemental material, sj-docx-1-ped-10.1177_17579759241293453 for Galvanising social innovation in gambling harms reduction: a process evaluation of a multi-component Community of Practice by Thomas Mills, Jo Evans, Catherine L. Jenkins, James Grimes, Paula Reavey, Jane Wills and Susie Sykes in Global Health Promotion

sj-docx-2-ped-10.1177_17579759241293453 – Supplemental material for Galvanising social innovation in gambling harms reduction: a process evaluation of a multi-component Community of Practice Supplemental material, sj-docx-2-ped-10.1177_17579759241293453 for Galvanising social innovation in gambling harms reduction: a process evaluation of a multi-component Community of Practice by Thomas Mills, Jo Evans, Catherine L. Jenkins, James Grimes, Paula Reavey, Jane Wills and Susie Sykes in Global Health Promotion

## References

[1] Lave J Wenger E. Situated Learning: Legitimate Peripheral Participation. New York, NY: Cambridge University Press; 1991.

[bibr2-17579759241293453] Hennein R Ggita JM Turimumahoro P Ochom E Gupta AJ Katamba A , et al. Core components of a Community of Practice to improve community health worker performance: a qualitative study. Implement Sci Commun. 2022; 3: 27. 35272705 10.1186/s43058-022-00279-1 PMC8908651

[3] European Commission. The Communities of Practice Playbook. European Commission Joint Research Centre. Luxembourg: Publications Office of the European Union; 2021.

[4] Del Fabbro L Rowe Minniss F Ehrlich C Kendall E . Political challenges in complex place-based health promotion partnerships: lessons from an exploratory case study in a disadvantaged area of Queensland, Australia. Int Q Community Health Educ. 2016; 37: 51–60. 28038500 10.1177/0272684X16685259

[5] Barnes C Sutherland R Jones G Kingon N Wolfenden L. Development and piloting of a Community of Practice to support learning and improvement in health promotion practice within NSW local health districts. Public Health Res Pract. 2023; 33. 10.17061/phrpp3332326 37699767

[6] Barbour L Armstrong R Condron P Palermo C. Communities of practice to improve public health outcomes: a systematic review. J Knowl Manag. 2018; 22: 326–343.

[7] Ranmuthugala G Plumb JJ Cunningham FC Georgiou A Westbrook JI Braithwaite J. How and why are communities of practice established in the healthcare sector? A systematic review of the literature. BMC Health Serv Res. 2011; 11: 273. 21999305 10.1186/1472-6963-11-273 PMC3219728

[8] South J Bagnall AM Stansfield JA Southby KJ Mehta P. An evidence-based framework on community-centred approaches for health: England, UK. Health Promot Int. 2019; 34: 356–366. 29206912 10.1093/heapro/dax083 PMC6445340

[9] Estacio EV Oliver M Downing B Kurth J Protheroe J. Effective partnership in community-based health promotion: lessons from the health literacy partnership. Int J Environ Res Public Health. 2017; 14: 1550. 10.3390/ijerph14121550 PMC5750968 29232877

[10] Goyder E Blank L Baxter S van Schalkwyk MC. Tackling gambling related harms as a public health issue. Lancet Public Health. 2020; 5: e14–e15. 10.1016/S2468-2667(19)30243-9 31831371

[11] Tucker JD Manderson L Amazigo U Alger J Chen E Labarda M , et al. Social innovation in health: concepts and practice. BMJ Innov. 2022; 8: 133–136.

[12] Whitty M Breen H Paterson M Sollis K. Health promotion strategies to address gambling-related harm in indigenous communities: a review of reviews. Crit Gambl Stud. 2021; 2: 39–54.

[13] Jenkins CL Mills T Bland C Grimes J Reavey P Wills J , et al. Involving Lived Experience in regional efforts to address gambling-related harms. BMC Pub Health. 2024; 24: 384. 38317155 10.1186/s12889-024-17939-7 PMC10840217

[14] Skivington K Matthews L Simpson SA Craig P Baird J Blazeby JM , et al. Framework for the development and evaluation of complex interventions: gap analysis, workshop and consultation-informed update. Health Technol Assess. 2021; 25: 1–132. 10.3310/hta25570 PMC7614019 34590577

[15] Davidoff F Dixon-Woods M Leviton L Michie S. Demystifying theory and its use in improvement. BMJ Qual Saf. 2015; 24: 228–238. 10.1136/bmjqs-2014-003627 PMC4345989 25616279

[16] Malterud K Siersma VD Guassora AD. Sample size in qualitative interview studies: guided by information power. Qual Health Res. 2016; 26: 1753–1760. 26613970 10.1177/1049732315617444

[17] Mills T Lawton R Sheard L. Advancing complexity science in healthcare research: the logic of logic models. BMC Med Res Methodol. 2019; 19: 55. 30871474 10.1186/s12874-019-0701-4 PMC6419426

[18] NHSE. Partnership working between voluntary, community, faith and social enterprise (VCFSE) sector organisations and ICSs to improve health and care outcomes. NHS England [Internet]. 2023 [cited 2023 December 19]. Available from: https://www.england.nhs.uk/long-read/partnership-working-between-voluntary-community-faith-and-social-enterprise-vcfse-sector-organisations-and-icss-to-improve-health-and-care-outcomes/

[19] South J Stansfield J . Asset-based public health – shifting evidence and practice [Internet]. Local Government Association; 2020 [cited 2023 November 1]. Available from: https://www.local.gov.uk/asset-based-public-health-shifting-evidence-and-practice

[20] Mills T Grimes J Caddick E Jenkins CL Evans J Moss A , et al. ‘Odds Are: They Win’: a disruptive messaging innovation for challenging harmful products and practices of the gambling industry. Public Health. 2023; 224: 41–44. 37714065 10.1016/j.puhe.2023.08.009 PMC10627150

