# Supplementary figures and images for: Galvanising social innovation in gambling harms reduction: a process evaluation of a multi-component Community of Practice
Source: Glob Health Promot. 2024 Nov 24;32(3):48–57. doi: 10.1177/17579759241293453 (PMC12521753; doi:10.1177/17579759241293453)

***Supplementary file 1***


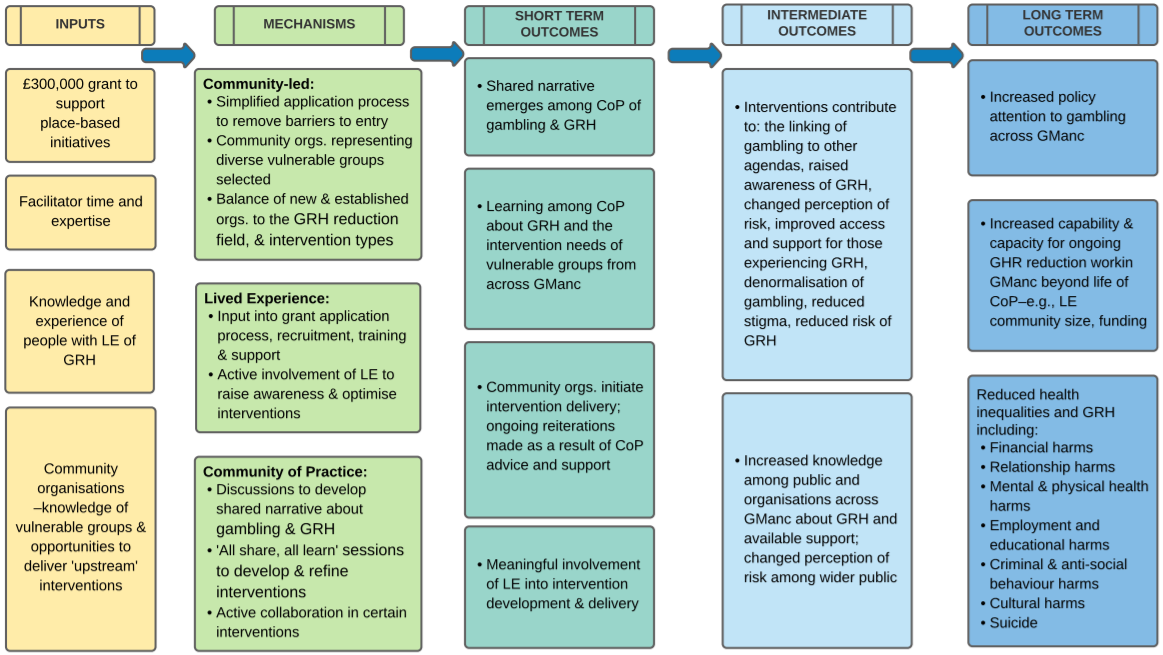

Supplement: sj-docx-1-ped-10.1177_17579759241293453 – Supplemental material for Galvanising social innovation in gambling harms reduction: a process evaluation of a multi-component Community of Practice [file sj-docx-1-ped-10.1177_17579759241293453.docx]
