# Supplementary material for: Galvanising social innovation in gambling harms reduction: a process evaluation of a multi-component Community of Practice
Source: Glob Health Promot. 2024 Nov 24;32(3):48–57. doi: 10.1177/17579759241293453 (PMC12521753; doi:10.1177/17579759241293453)
Supplement: sj-docx-2-ped-10.1177_17579759241293453 – Supplemental material for Galvanising social innovation in gambling harms reduction: a process evaluation of a multi-component Community of Practice [file sj-docx-2-ped-10.1177_17579759241293453.docx]

***Supplementary file 2***

CAGH Community Projects: Learning Reflections

*Information can be captured in any or multiple formats, e.g. video, presentation, writing – it is not intended to be a lengthy report and try to make it as engaging as possible to help reach a new audience with this learning. Please provide evidence, e.g. links to external content such as media coverage, resources and materials generated, etc. to enable us to understand the impact of the project and share outputs with a wider audience.*

1. Who are you and what does your organisation do?
2. What was the problem that your project sought to address and how did your project seek to do this?
3. What challenges / barriers did you face in delivering your project? Were you able to overcome these? How?
4. What enabled / helped you to deliver your project? *(consider things within your organisation, e.g. “we have good links with…” or “we have been working on gambling for X years so knew…” and external factors as part of the wider ‘system’, e.g. “we got good support from Y”)*
5. What was the impact of your project… *(please provide case stories to illustrate any numbers quoted, e.g., “we reached 80 people, this is Mo’s experience of this”)*

- For direct beneficiaries? (Who? How many? In what way?)
- On your organisation?
- On the wider system? (Think about wider networks? Connections? Embedding gambling in other areas?)

1. If you were to run the same project again, what (if anything) would you do differently?
2. What do you think others can learn from your project? *(this might be practical things, like how to implement your initiative elsewhere, or contributions to knowledge and understanding of gambling harms in Greater Manchester)*
3. Does your organisation have any plans to continue this project in any form? *(this might be continuing the existing project as it is, or using outcomes from your project to inform other services or initiatives)*
4. What are your reflections on the value of the Community of Practice? Do you have any recommendations for how this could operate in the future? Do you have any recommendations for the wider GM system and / or partners? *(please be specific)*
5. Is there anything else you would like to share that hasn’t been captured elsewhere?
